# Optimal Pharmacologic Treatment Strategies in Obesity and Type 2 Diabetes

**DOI:** 10.3390/jcm3020595

**Published:** 2014-06-18

**Authors:** Gayotri Goswami, Nataliya Shinkazh, Nichola Davis

**Affiliations:** 1 North Bronx Healthcare Network, 3424 Kossuth Avenue, Bronx, NY 10467, USA; E-Mail: nichola.davis@nbhn.net; 2 Touro College of Pharmacy, New York, NY 10027, USA; E-Mail: shinkazh.n@gmail.com

**Keywords:** obesity, type 2 diabetes, obesity, obese diabetics, weight neutral diabetes medications, anti-obesity drugs, weight loss in diabetes

## Abstract

The prevalence of obesity has increased to pandemic levels worldwide and is related to increased risk of morbidity and mortality. Metabolic comorbidities are commonly associated with obesity and include metabolic syndrome, pre-diabetes, and type 2 diabetes. Even if the prevalence of obesity remains stable until 2030, the anticipated numbers of people with diabetes will more than double as a consequence of population aging and urbanization. Weight reduction is integral in the prevention of diabetes among obese adults with pre-diabetes. Lifestyle intervention and weight reduction are also key in the management of type 2 diabetes. Weight loss is challenging for most obese patients, but for those with diabetes, it can pose an even greater challenge due to the weight gain associated with many treatment regimens. This article will review optimal treatment strategies for patients with comorbid obesity and type 2 diabetes. The role of anti-obesity agents in diabetes will also be reviewed. This literature review will provide readers with current strategies for the pharmacologic treatment of obesity and diabetes with a focus on the weight outcomes related to diabetes treatments.

## 1. Introduction

Excess body fat or obesity defined as a body mass index (BMI) ≥ 30 kg/m^2^, is an important factor in the pathogenesis of insulin resistance and substantially increases the risk of type 2 diabetes. Weight reduction is integral in the prevention of diabetes among obese adults with pre-diabetes. In obese patients with diagnosed type 2 diabetes, weight reduction of 5%–10% from baseline can lead to improved glucose control as well as improvements in blood pressure and cholesterol [1]. Unfortunately, many available medications for type 2 diabetes promote weight gain, thus making weight loss in this population challenging. In the past 10 years, the treatment of diabetes and obesity has evolved with the discovery of novel pathways in the pathophysiology of diabetes. Additionally, newly approved medications in the treatment of obesity can lead to improvements in glucose control in those with diabetes. Newer treatment strategies may allow practicioners to treat diabetes in ways that will not worsen obesity outcomes. This article will review the pathophysiology of diabetes focusing on treatment strategies that target physiologic pathways which results in weight neutral or weight loss effects. Further we will review the impact of FDA approved obesity medications on diabetes control.

The prevalence of obesity has sharply increased in the last decades of the 20th century and has reached epidemic proportions in the United States. Globally, the health burden from obesity now exceeds that of hunger, and with the exception of those in sub-Saharan Africa almost every country faces alarming obesity rates [2]. Overweight status and obesity fuel the global diabetes epidemic and if current trends continue, the number of overweight people (BMI ≥ 25 kg/m^2^) is projected to increase from 1.3 billion in 2005 to nearly 2.0 billion by 2030 [3]. Estimates suggest that by 2030, diabetes will affect 438 million people worldwide. These increases are thought to be in part due to increases in nutrition transitions and decreases in physical activity promoting positive energy balance. Rapid urbanization and transitions in nutritional status has led to Asia accounting for 60% of the world’s diabetic population [4,5].

## 2. Development of Type 2 Diabetes

Obesity is the most common cause of insulin resistance, however mechanisms through which obesity leads to insulin resistance are still not completely understood [6]. The most logical explanation is that there are changes in the environment which create positive energy balances and leads to an expansion of adipose tissue in genetically predisposed individuals. It has become increasingly evident, that adipose tissue actively secretes peptides that contribute to the development of insulin resistance. Adipokines are a variety of bioactive peptides secreted and expressed by adipose tissue that act at both the local and systemic level. These include hormones such as adiponectin, leptin, and resistin which have dramatic effects on glucose metabolism and insulin action [7]. Additionally, circulating levels of pro-inflammatory molecules such as tumor necrosis factor-α, interleukin-6, transforming growth factor-β, retinol binding protein-4, C-reactive protein, and monocyte chemotactic protein-1 are increased in obesity [8,9]. These pro-inflammatory molecules are believed to induce systemic insulin resistance contributing to the pathogenesis of many of the metabolic complications of obesity including type 2 diabetes [10]. Further, elevated levels of plasma free fatty acids (FFA) are seen in obese subjects with insulin resistance due to enhanced lipolysis. This leads to an increase in cellular FFA uptake and stimulation of lipid oxidation. In the muscle, increased FFA oxidation leads to reduced insulin mediated glucose disposal, while in the liver, it stimulates gluconeogenesis and increases hepatic glucose output [11].

Prospective studies have revealed that insulin resistance predates the onset of type 2 diabetes by 10–20 years and it is the best clinical predictor of subsequent development of type 2 diabetes [12,13]. In the setting of insulin resistance, two important pathological defects lead to type 2 diabetes which include: (1) decreased ability of insulin to act on peripheral tissues to stimulate glucose metabolism or inhibit hepatic glucose output; and (2) inability of the pancreatic beta cells to fully compensate for the degree of insulin resistance in genetically predisposed individuals, leading to a state of relative insulin deficiciency [11,14,15]. In the absence of a defect in beta cell function individuals can compensate for insulin resistance with appropriate hyperinsulinemia and maintain glucose homeostasis [16]. Type 2 diabetes is thus characterized by both insulin resistance and insulin deficiency, the former being manifested both in the liver and peripheral tissues. Hepatic insulin resistance results in overproduction of glucose in the basal state, despite the presence of fasting hyperinsulinemia with fasting hyperglycemia as the clinical end product. Insulin resistance of peripheral tissues, predominantly muscle, results in impaired insulin stimulated glucose uptake and postprandial hyperglycemia.

## 3. Treating Type 2 Diabetes

**Figure 1:**
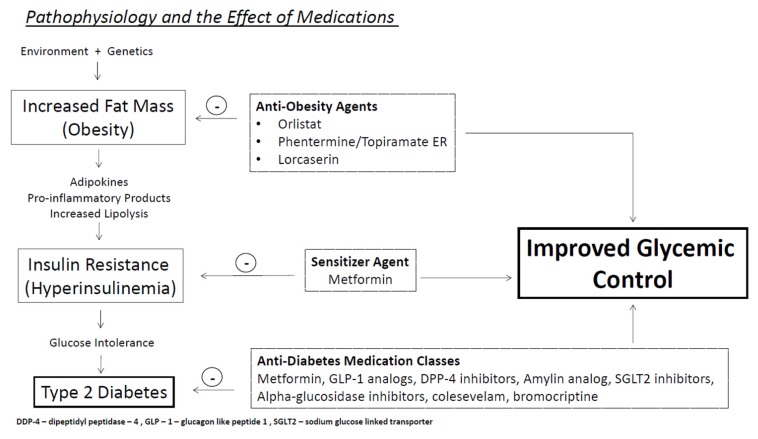
Pathophysiology and the Effect of Medications.

Lifestyle intervention with diet, exercise and important weight loss strategies are integral in the treatment of type 2 diabetes [17]. A full discussion on the role of lifestyle intervention in diabetes mellitus treatment while important is beyond the scope of this article. This article will focus on pharmacologic therapy of obesity and type 2 diabetes (see Figure 1). Medication therapy for type 2 diabetes ranges from monotherapy or combination therapy with oral antidiabetic agents to physiologic insulin replacement therapy [17,18]. In treating the obese patient with type 2 diabetes, it is important to consider treatment regimens that do not worsen weight status. Unfortunately, many effective agents that reduce glucose cause weight gain. Secretagogues which include sulfonylureas (glyburide, glipizide, glimepiride) and non-suflonylureas (repaglinide, nateglinide) stimulate pancreatic beta cell function and increase circulating insulin levels. Both classes have significant glycosylated hemoglobin (HbA1c) lowering capacity. Sulfonylureas on average lower HbA1c levels by 1.5%–2%, and non-sulfonylureas are slightly less effective and lower HbA1c levels by up to 1% [19]. Average weight gain associated with secretagogue therapy is 2–3 kg [20]. Thiazolidinediones (rosiglitazone, pioglitazone) work through binding of the peroxisome proliferator activator receptor-ƴ (PPAR-ƴ), and thereby enhance insulin sensitivity at the liver, fat, and muscle tissues. These agents lower HbA1c levels by approximatey 1.5% but their full effect can take up to 4 months, and weight gain associated with thiazolidinediones can range from 1.5 to 4 kg [21,22]. Insulin, considered the most effective treatment for lowering HbA1c causes an increase in weight of up to 1.8 kg with every 1% HbA1c reduction from baseline [23]. However some therapies are weight neutral or cause weight loss, making them advantageous in patients who are overweight or obese (see Table 1).

**Table 1:** Anti-diabetic agents with beneficial effects on weight.

Class & Medication	Trade Name	Dosage Availability	Weight Effect	A1c Reduction	Side Effects	Cost *	Notes & Considerations
Biguanide Metformin	Glucophage™ Glucophage ER™	*Oral tablet* 500/850/1000 mg 500/750/1000 mg	Weight loss [24,25] 2–3 kg	1.5%–2% [24,25]	GI ^1^, lactic acidosis	$0.70–1.44 $0.75–1.03 per tablet	First Line agent; Avoid in renal dysfunction
GLP 1 analogs Exenatide Liraglutide	Byetta™/Bydureon™ Victoza™	*Subcutaneous* 5–10 mcg 0.6/1.2/1.8 mg	Weight loss [26] 2.87–3.84 kg	0.9%–1% [27,28,29,30,31,32,33]	*Class Effects:* GI, acute pancreatitis, medullary thyroid cancer	Byetta™/Bydureon™—$197/$122 Victoza™—$71.42 (unit) $428 (package)	Injectable; Avoid in renal dysfunction & history of pancreatitis
DPP 4 inhibitors Sitagliptin Saxagliptin Linagliptin Alogliptin	Januvia™ Onglyza™ Tradjenta™ Nesina™	*Oral tablet* 25/50/100 mg 2.5/5 mg 5 mg 6.25/12.5/25 mg	Weight neutral [34]	0.7%–1% [34]	*Class Effects:* URI ^2^, anaphylaxis, acute pancreatitis	$11.35 $11.16 $11.35 $11.35 per tablet	Oral formulation; Renal dose adjustments needed; Avoid when history of pancreatitis
Amylin analog Pramlintide	Symlin™	*Subcutaneous* 30/60/120 mcg	Weight loss [35] 2.57 kg	0.3%–0.4% [35]	GI	$357.35 (120 mcg) $292.56 (60 mcg) (package)	Indicated for insulin dependent patients; Only used in the setting of insulin co-administration
SGLT2 inhibitors Canagliflozin Dapagliflozin	Invokana™ Farxiga™	*Oral tablet* 100/300 mg 5/10 mg	Weight loss [36,37,38] 2–3 kg	0.6%–0.9% [39]	*Class Effects:* Genital mycotic infection, UTI ^3^, increase in LDL-C ^4^, serum creatinine and K+ ^5^	Invokana™ $11.57 per tablet Farxiga™—NA ^6^	Extensive side effect profile
Miscellaneous Agents Acarbose/Miglitol Colesevelam Bromocriptine	Precose™/Glyset™ Welchol™ Cycloset™	*Oral tablet* 25/50/100 mg 625 mg 1.6–4.8 mg	Weight loss—1.1 kg [40] Weight neutral [41,42,43,44] Weight neutral [45,46,47,48]	1% [40] 0.5% [41,42,43,44] 0.6%–0.9% [45,46,47,48]	GI GI GI, asthenia, HA ^7^	$0.89/0.97/1.16 $2.30 (625 mg) $2.75 per tablet	Colesevelam CI ^8^ when TG ^9^ > 500 mg/dL; Bromocriptine CI in uncontrolled HTN ^10^, syncopal migraine, breastfeeding women

^1^ GI—gastrointestinal; ^2^ URI—upper respiratory tract infection; ^3^ UTI—urinary tract infection; ^4^ LDL-C—low density lipoproteins cholesterol; ^5^ K+—potassium; ^6^ NA—not available; ^7^ HA—headache; ^8^ CI—contraindicated; ^9^ TG—triglycerides; ^10^ HTN—hypertension; * Prices represent average wholesale price (AWP) referenced from a combination of resources which include: (1) Thomson Reuters Micromedex Clinical Evidence Solutions. Thomson Reuters; c2011. RED BOOK Drug References; c2011 (cited 20 January 2014); (2) Lexicomp Online™, Drug Information™, Hudson, Ohio: Lexi-Comp, Inc. 2014; 20 January 2014.

## 4. Medications for Type 2 Diabetes with Weight Neutral or Weight Loss Effects

### 4.1. Biguanide Medications

#### Metformin (Glucophage, Glucophage XR)

Metformin is the first line agent of choice as it reduces insulin resistance and may reduce weight, but due to intolerable side effects and many contraindications, its use is sometimes limited. Metformin is FDA approved for the treatment of type 2 diabetes. Additionally, the current American Diabetes Association (ADA) guidelines recommend its use in the prevention of type 2 diabetes in patients that have impaired glucose tolerance, impaired fasting glucose, or HbA1c levels of 5.7%–6.4%, especially in patients with a BMI > 35 kg/m^2^, <60 years of age, or women with prior gestational diabetes [49]. Metformin is of the biguanide class of antidiabetic oral agents and acts to improve glycemic control by reducing hepatic glucose production, increasing intestinal glucose absorption, and increasing insulin sensitivity at the hepatic and peripheral tissues. Metformin reduces HbA1c levels by 1.5%–2%, with positive effects on the lipid panel. Metformin does not increase insulin secretion from the pancreatic beta-cells and hypoglycemia and weight gain are minimal. Metformin is indicated as the first line agent for obese patients with diabetes, and although metformin is considered to have weight neutral effects, some studies have shown weight loss of up to 2–3 kg [24,25]. Metformin side effects primarily include transient gastrointestinal symptoms, but 4% of patients must cease metformin therapy due to poor tolerance [25]. Metformin is also contraindicated in patients with renal impairment, congestive heart failure, and relatively contraindicated in patients predisposed to metabolic acidosis. As a result of its side effect profile and contraindications, metformin therapy is not indicated for a substantial proportion of the population, and alternative treatment options to achieve satisfactory glycemic control without additional weight gain must be identified and considered when treating obese diabetic patients.

### 4.2. Role of Incretin Hormones

Glucose homeostasis is a complex interplay of multiple hormones: insulin and amylin produced by the beta cells, glucagon produced by pancreatic alpha cells and the incretin hormones; glucagon-like peptide 1 (GLP-1) and glucose insulinotropic polypeptide (GIP), and dipeptidyl peptidase-4. GLP-1 has gained much attention in the past decade due to its effect on glucose control through several mechanisms including enhancement of glucose-dependent insulin secretion, slowing of gastric emptying, regulation of postprandial glucagon and reduction of food intake. The incretin effect, in which oral glucose has a greater stimulatory effect on insulin secretion than intavenous glucose illustrates the role of GLP-1 in glucose homeostasis [50]. This incretin effect has been shown to be decreased in adults with type 2 diabetes when compared to normal subjects and is most likely due to decreased levels of GLP-1 [51]. GLP-1 is produced from the proglucagon gene in L-cells of the small intestine and is secreted in response to nutrients and exerts its main effect by stimulating glucose—dependent insulin release from the pancreatic islets [52]. GLP-1 has a short half life and is rapidly degraded to its metabolites by dipeptidyl peptidase-4 enzyme (DPP-4) [53].

#### 4.2.1. GLP-1 Analogs

##### Exenatide (Byetta, Bydureon), Liraglutide (Victoza)

Exenatide and Liraglutide are FDA approved for the treatment of type 2 diabetes. These agents are recommended as first line therapy in obese diabetic patients who have a contraindication or cannot tolerate metformin therapy [17]. Exenatide is a synthetic analog of exendin-4, and liraglutide is an acylated human GLP-1 receptor agonist. Exenatide is formulated in injectable immediate and extended release products. The immediate release formulation marketed as Byetta, is administered twice daily before meals and the extended release formulation, Bydureon, is administered once weekly. Exenatide is most effective for postprandial glucose control, reducing postprandial glucose and HbA1c levels by approximately 50 mg/dL, and 0.8%, respectively, with an average weight reduction of 0.7–2.5 kg [27,28,29].

Liraglutide is injected once daily and has shown a maximal HbA1c reduction of approximately 1.14% [30,31,32,33]. When compared to exenatide immediate release, liraglutide reduced HbA1c and fasting plasma glucose levels significantly more than exenatide by 0.33% and 18 mg/dL, respectively, but exenatide was more effective at reducing postprandial glucose levels. Both medications have similar effects on weight reductions with 3.24 kg lost with liraglutide and 2.87 kg lost with exenatide [26].

In short-term clinical trials, GLP-1 analogs have been shown to be effective at inducing weight loss in obese adults without diabetes. When studied for 24 weeks, obese, non-diabetic patients receiving exenatide lost 5.1 kg from baseline *versus* 1.6 kg with placebo [54]. In another study, after 16 weeks of treatment with no lifestyle intervention, non-diabetic obese women lost a mean of 2.49 kg with exenatide treatment as compared with 0.43 kg of weight gain with placebo [55]. Liraglutide was studied in obese patients without type 2 diabetes for 20 weeks and was shown to be more effective at inducing weight loss than orlistat or placebo, with a mean dose dependent weight loss that ranged from 4.8 to 7.2 kg [56]. While these clinical trials are promising, currently there are no recommendations to treat obesity with GLP-1 analogs in the absence of diabetes.

Frequent side effects of GLP-1 analogs are of gastrointestinal origin, and include nausea and vomiting. Although these side effects decrease over time, the medications in this class require slow dose titration for improved tolerance. GLP-1 analogs increase the odds of pancreatitis two fold, and are therefore contraindicated in patients with a history of pancreatitis [57]. Exenatide is contraindicated in renal dysfunction with creatinine clearance less than 30 mL/min. Additionally, this class of medications has black box warnings and is contraindicated in patients with thyroid c-cell tumors, a family history of medullary thyroid carcinoma and multiple endocrine neoplasia syndrome type 2.

#### 4.2.2. Dipeptidyl Peptidase 4 (DPP-4) Inhibitors

##### Sitagliptin (Januvia), Saxagliptin (Onglyza), Linagliptin (Tradjenta), Alogliptin (Nesina)

DPP-4 inhibitors are a class of oral antidiabetic agents that also target the incretin system to improve glycemic control. These medications work by inhibiting the degradation of endogenous GLP-1 and glucose-dependent insulinotropic polypeptide (GIP), and thereby increase insulin release and decrease glucagon levels via glucose dependent means. As monotherapy, DPP-4 inhibitors effectively reduce fasting, postprandial, and HbA1c levels by 0.7%–1%, with minimal hypoglycemia, and a weight neutral effect. In obese patients, when sitagliptin was added to metformin therapy, patients experienced statistically significant weight loss of 1.5 kg and significantly less hypoglycemia when compared to patients receiving metformin and glipizide who experienced a weight gain of 1.1 kg [34]. Though weight loss has not been established as a class-effect, DPP-4 inhibitors are safe and effective agents in the treatment of type 2 diabetes and are a viable option for obese patients, as they do not contribute to weight gain.

DPP-4 inhibitors are well tolerated, although post-marketing studies have shown that adverse effects can be serious and include anaphylaxis, angioedema, Stevens Johnson syndrome, and acute pancreatitis. All medications in this class except linagliptin require renal dose adjustments.

### 4.3. Role of Amylin

Amylin, a peptide stored in the pancreatic beta cells, is co-secreted with insulin and has complemetary actions in regulating nutrient levels. Amylin is deficient in type 1 diabetes and relatively deficient in insulin requiring type 2 diabetes. It affects glucose control by slowing gastric emptying, suppressing postprandial glucagon secretion, and reducing food intake, but unlike GLP-1 it does not stimulate insulin secretion. Amylin and GLP-1 appear to differ in their magnitudes of physiologic effects as they bind to different receptors in the area postrema—the part of the brain that may be key for effects on satiety [58,59]. Pramlinitide, an injectable amylin analog, is FDA approved for the treatment of type 1 diabetes and insulin treated type 2 diabetes and administered subcutaneously at mealtimes.

#### Pramlintide (Symlin)

Pramlintide is indicated in patients who use mealtime insulin but are still unable to achieve glycemic control. Sulfonylurea agents or metformin may be used in conjunction with insulin and pramlintide in the treatment of type 2 diabetes. Pramlintide is a separate injection given prior to meals, and its predominant effect is on postprandial glucose levels. In obese patients with type 2 diabetes, a meta-analysis found that pramlintide significantly reduced HbA1c levels by 0.3%–0.42% and weight by 2.57 kg, respectively [35]. Gastrointestinal adverse effects occur with initiation of therapy and include nausea and vomiting, but subside over time. When combined with insulin, hypoglycemia is a major adverse effect and should be prevented by empiric dose reduction of mealtime insulin by 30%–50%. Due to minimal efficacy and the need for frequent injection, pramlintide is not recommended as first line therapy by the ADA or European Association for the Study of Diabetes (EASD), but because of its positive effects on weight and the reduced insulin requirements, it may be considered as an option for obese type 2 diabetic patients that use mealtime insulin [17].

### 4.4. Role of Sodium-Glucose Linked Transporter 2 Inhibition

Sodium-glucose linked transporter 2 (SGLT2) inhibitors are a novel class of oral antidiabetic agents newly approved by the FDA for the treatment of type 2 diabetes. Hyperglycemia increases the filtered load of glucose at the glomerulus and the bulk of the filtered load is reabsorbed by the low affinity/high capacity sodium glucose co-transporter—SGLT2 located in the S1 segment of the proximal tubule [60]. Residual glucose is then absorbed by the high-affinity/low capacity SGLT1 in the S3 segment of the loop of Henle and the distal tubule [60]. In patients with diabetes, elevated blood glucose levels lead to an increase in the glucose load filtered by the glomerulus. This increase in maximum tubular glucose transport is attributed to up-regulation of SGLT2 and therefore SGLT2 inhibitors would induce greater levels of urinary glucose excretion in patients with diabetes compared to individuals with normal glucose metabolism [61,62].

#### Canagliflozin (Invokana), Dapagliflozin (Farxiga)

The glucose lowering effect of SGLT2 inhibitors is independent of insulin and is limited by the filtered load of glucose and osmotic diuresis. These agents may be beneficial in the obese diabetic due to the weight loss observed secondary to the caloric loss. The glucose excreted in the urine due to SGLT2 inhibition translates to about 200–300 calories each day and a weight loss of 2–3 kg has been demonstrated with SGLT2 inhibitors as monotherapy and in combination with metformin [36,37,38]. Significant reduction in waist circumference has also been demonstrated which would be consistent with a reduction in fat mass [63].

There is absence of long term efficacy and safety data on SGLT2 inhibitors. Completed trials have shown benefits in both fasting and postprandial glucose measures with a lowering of HbA1c between 0.6% (Dapagliflozin dose of 2.5 mg) to 0.9% (Canagliflozin 300 mg) [39]. Adverse effects of this drug class can be extensive; in the genitourinary system these medications cause genital mycotic and urinary tract infections, vulvovaginal pruritis, and increased urination. Other adverse effects include thirst, dry mouth, polydipsia, constipation, and nausea. These agents have also been shown to increase serum creatinine and LDL-cholesterol levels, as well as cause hyperkalemia in patients with renal impairment or who are taking potassium sparing medications [64]. SGLT2 inhibitors are not indicated in patients with renal impairment, additionally, canagliflozin increases digoxin concentration levels, and because both of these medications are mediated via UDP-glucuronosyltransferase (UGT)—metabolism, drug interactions must be monitored closely when these agents are prescribed.

### 4.5. Alpha-Glucosidase Inhibitors

#### Acarbose (Precose), Miglitol (Glyset)

Alpha-glucosidase inhibitors are oral antidiabetic agents indicated for the treatment of type 2 diabetes. They competitively inhibit intestinal enzymes and thereby delay the breakdown of sucrose and complex carbohydrates. This class of agents is more effective at reducing post prandial glucose, which results in a maximal HbA1c reduction of 1%. Alpha-glucosidase inhibitors are considered weight neutral, and a recent observational study found that patients lost 1.1 kg while on acarbose therapy [40]. These medications must be administered with the first bite of food prior to every meal. As monotherapy, they do not cause hypoglycemia, but patients must be warned to treat any occurrences of hypoglycemia with oral glucose, rather than complex carbohydrates. Side effects are primarily of gastrointestinal origin and include flatulence, bloating abdominal discomfort and diarrhea, which often limit the use of these medications. Alpha-glucosidase inhibitors are not recommended as first line agents in the treatment of diabetes primarily because of their side effect profile together with minimal effectiveness, but they may be considered as a viable option in obese diabetic patients who want to avoid weight gain.

### 4.6. Miscellaneous Agents

#### 4.6.1. Colesevelam (Welchol)

Colesevelam is a bile acid sequestrant which is indicated for the treatment of type 2 diabetes. Its mechanism of action in improving glycemic control is unknown. In clinical trials, when used in combination with metformin, sulfonylurea, or insulin-based treatment, colesevelam provided additional HbA1c reductions of approximately 0.5%, with no evidence of increased rates of hypoglycemia or weight gain [41,42,43,44]. Bile acid sequestrants are an effective treatment option for hyperlipidemia, and additional benefits of colesevelam in patients with type 2 diabetes include reductions in LDL and total cholesterol levels, and increases in HDL cholesterol levels. Side effects of colesevelam are predominantly of gastrointestinal origin and may limit its use. It is contraindicated in patients with a history of bowel obstruction, hypertriglyceridemia-induced pancreatitis, or triglycerides levels >500 mg/dL as this drug class increases triglycerides levels.

#### 4.6.2. Bromocriptine (Cycloset)

Bromocriptine is typically used in the treatment of Parkinson’s disease as a dopamine agonist and works on post-synaptic dopamine receptors but is also indicated for the treatment of type 2 diabetes. Its mechanism of improved glycemic control is unknown. After 6 months of treatment, obese patients with uncontrolled type 2 diabetes receiving bromocriptine as add on therapy experienced HbA1c reductions ranging between 0.6%–0.9% [45,46,47,48]. Major side effects are mild and temporary, and include nausea, asthenia, constipation, dizziness, and rhinitis. Additional hypoglycemia or weight gain with bromocriptine was not shown in any clinical trials. Bromocriptine is contraindicated in uncontrolled hypertension, syncopal migraine, or breastfeeding women.

## 5. Anti-Obesity Agents

Targeting weight primarily, can also lead to further reductions in glucose. Thus it is important to treat obesity in addition to treating diabetes. Three medications are currently approved by the FDA for long-term treatment of obesity. Studies of these medicines in patients with diabetes also show an improvement in glycemic control. The following section will summarize medicines approved for obesity and the clinical trial data highlighting their impact on glucose control.

### 5.1. Orlistat (Alli, Xenical)

Orlistat is indicated for long term treatment of obese patients with a BMI of ≥30 kg/m^2^ or overweight patients with a BMI of ≥27 kg/m^2^ with a concomitant comorbidity like hypertension, hyperlipidemia, or diabetes. Orlistat induces weight loss by blocking dietary absorption of fat. Its mechanism of action is to inhibit gastric and pancreatic lipase enzymes with limited systemic absorption. Combined with diet and exercise in the first year of treatment of obese patients, orlistat 120 mg three times daily achieved significantly greater weight reduction as compared to placebo (8.8% *versus* 5.8%, *p* < 0.001, respectively) at 1 year. After 2 years of treatment, patients lost 7.6% of their total body weight from baseline as compared to placebo with a weight loss of 4.2% [65]. In patients without diabetes, orlistat was found to reduce weight by a mean of 2.8 kg at 4 years, and reduce progression to diabetes [66]. In adults with poorly controlled diabetes, as compared to placebo, orlistat significantly reduced weight by 2.9 kg more (*p* < 0.001), and improved glycemic control with lowering of HbA1c by 0.29% (*p* = 0.014) and fasting plasma glucose levels by 24 mg/dL (*p* = 0.001). Additionally, orlistat treatment showed greater reduction in systolic blood pressure, LDL and total cholesterol levels [67].

Common side effects observed with orlistat are primarily of gastrointestinal origin and may lead to discontinuation of treatment. Orlistat is contraindicated in pregnancy, patients with chronic malabsorption syndrome, and cholestasis. Additionally, due to its mechanism of action, orlistat reduces the absorption of fat-soluble vitamins and may interact with various medications. Patients taking orlistat should be advised to supplement with multivitamins to guarantee sufficient nutrition and speak to their doctor prior to starting therapy to avoid drug interactions.

### 5.2. Phentermine/Topiramate Extended Release (Qsymia)

The combination dosage form of phentermine hydrochloride with topiramate extended release (ER) was approved by the Food and Drug Administration (FDA) in 2012 as an adjunct to diet and exercise in obese and overweight adults, with at least one weight-related comorbidity for chronic weight management. Topiramate, an anticonvulsant indicated for the treatment of partial seizures and migraine prophylaxis, has been shown to increase weight loss in obese patients. Due to the increased risk of congenital malformations with topiramate, the FDA requires a Risk Evaluation and Mitigation Strategy (REMS) for the drug combination.

After 1 year of treatment with phentermine/topiramate, combined with diet, patients with a BMI ≥ 35 kg/m^2^ achieved significantly more weight loss as compared to placebo (5.1% with 3.75/23 mg (*n* = 241), 10.9% with 15/92 mg (*n* = 512), 1.6% with placebo (*n* = 514) [68]. Additionally, more patients lost at least 5% weight from baseline in the phentermine/topiramate arms (44.9% with 3.75/23 mg, 66.7% with 15/92 mg, and 17.3% with placebo).

In the 1 year CONQUER study, patients with a BMI of 27–45 kg/m^2^ and two or more comorbidities achieved significant weight loss compared to placebo (7.8% with 7.5/46 mg, 9.8% with 15/92 mg, 1.2% with placebo). Significantly more patients lost at least 5% weight from baseline in the phentermine/topiramate arms (62% in the 7.5/46 mg dose, 70% in the 15/92 mg dose, and 21% in the placebo group) [69].

The SEQUEL trial was a 1 year extension study of the patients from the CONQUER trial which evaluated long-term efficacy and safety of phentermine/topiramate ER regimens. Sustained weight loss from baseline was significant in the phentermine/topiramate groups as compared to placebo (9.3% with 7.5/45 mg, 10.5% with 15/92 mg, and 1.8% with placebo). Additionally, when compared with placebo, significantly more patients in each of the phentermine/topiramate groups achieved weight loss of ≥5%, ≥10%, ≥15%, and ≥20% [70]. In patients who had a diagnosis of type 2 diabetes at baseline, phentermine/topiramate reduced HbA1c by 0.4% and 0.2%, respectively with the 7.5/46 and 15/92 mg doses [70]. In a sub analysis of patients with pre-diabetes or metabolic syndrome, phentermine/topiramate significantly reduced progression to type 2 diabetes. In pre-diabetic patients, phentermine/topiramate reduced the incidence rate of type 2 diabetes by 48.6% with the 7.5/46 mg dose, and 88.6% with the 15/92 mg dose. In patients with metabolic syndrome, doses of 7.5/46 mg and 15/92 mg had a reduction of 76.6% and 79.7%, respectively [71]. At week 108, a significant increase in heart rate of 0.4 beats per minute (bpm), 1.3 bpm and 1.7 bpm in the placebo, 7.5/46 mg, and 15/92 mg subjects respectively, was noted. Additionally, patients treated with the phentermine/topiramate combination experienced greater reductions in serum bicarbonate from baseline, which may increase the risk of metabolic acidosis.

### 5.3. Lorcaserin (Belviq)

Lorcaserin was approved by the FDA in 2012 as an adjunct to diet and exercise for chronic weight management in obese or overweight adults with at least one weight-related comorbidity. Lorcaserin is thought to decrease food intake and promote satiety by selectively agonizing the serotonin 2C (5-HT_2c_) receptors on the propiomelanocortin neurons of the hypothalamus [72]. In a double-blind randomized trial, of 3182 obese and overweight adults, participants received lorcaserin 10 mg, or placebo twice daily for 1 year. At 1 year, 47.5% of patients in the lorcaserin group lost ≥5% body weight, or a mean loss of 5.8 kg as compared to 20.3% of placebo patients with a mean weight loss of 2.2 kg (*p* < 0.001) [73]. In the second year of follow-up, patients who had received lorcaserin were randomized to receive continued treatment or placebo; in year two of the study, patients who continued treatment with lorcaserin experienced slight weight regain while patients randomized to the placebo condition in year 2 had weight loss comparableto those receiving the placebo throughout the two year study period [73]. The BLOSSOM trial showed similar findings with more patients losing at least 5% of baseline weight with lorcaserin 10 mg once daily and twice daily regimens (40.2% and 47.2%, respectively) as compared with placebo (25%, *p* < 0.0001 for both regimens) [74].

The 1-year BLOOM-DM study randomized overweight and obese patients with type 2 diabetes treated with metformin, sulfonylurea, or both, and HgA1C levels between 7%–10% to receive lorcaserin 10 mg once daily, twice daily, or placebo [75]. More patients lost ≥5% body weight with lorcaserin once or twice daily as compared to placebo (44.7%, 37.5%, 16.1%, respectively, *p* < 0.001), with mean weight reduction of 5 kg with lorcaserin once daily, 4.7 kg with twice daily, and 1.6 kg with placebo (*p* < 0.001). HbA1C and fasting plasma glucose levels decreased significantly more in both lorcaserin groups as compared to placebo. HgA1C was reduced by 1.0% in the lorcaserin once daily group, 0.9% in the twice daily group, and 0.4% in the placebo group (*p* < 0.001). Rates of hypoglycemia were higher in the lorcaserin as compared to placebo group, occurring in 10.5% of the lorcaserin once daily group, and 7.4% in the twice daily group as opposed to 6.3% in the placebo group. No statistically significant differences between cholesterol or blood pressure levels were noted between any of the treatment groups.

Most common adverse effects of lorcaserin observed in clinical trials included upper respiratory infections, headache, dizziness, nasopharyngitis, and nausea. Rates of valvulopathy did not differ between any of the treatment arms. Lorcaserin is contraindicated in pregnancy as it may increase the risk of fetal harm. See Table 2 for a summary of the effect of anti-obesity drugs on glucose control.

**Table 2:** Effect of anti-obesity agents on glucose control.

Class & Medication	Trade Name	Dosage Availability	Weight Effect	A1c Reduction	Side Effects	Cost *	Notes & Considerations
Lipase Inhibitor Orlistat	Alli™ Xenical™	*Oral tablet* 60 mg 120 mg	Weight loss [65,66,67] 2.9 kg	0.29% [67]	GI ^1^	$0.59 (Alli™) $6.26 (Xenical™)	Avoid in liver disease
Appetite Suppressant Phentermine/Topiramate ER	Qsymia™	*Oral tablet* 3.75/23 mg 7.5/46 mg 11.25/69 mg 15/92 mg	Weight loss [68,69,70,71] 5.8kg	0.2%–0.4% [70]	Metabolic acidosis tachycardia, congenital malformations	$5.44 $6.15 $7.38 $7.98	Requires REMS ^2^ for congenital malformations; Sympathomimetic effects may exacerbate underlying cardiac disease
Serotonin-Receptor Agonist Lorcaserin	Belviq™	*Oral tablet* 10 mg	Weight loss [73,74,75] 5 kg	0.9%–1% [75]	GI, URI ^3^, HA ^4^, dizziness	$3.99	Drug interactions with serotonergic agents

^1^ GI—gastrointestinal; ^2^ REMS—risk evaluation and mitigation strategy; ^3^ URI—upper respiratory tract infection; ^4^ HA—headache; * Prices represent average wholesale price (AWP) referenced from a combination of resources which include: (1) Thomson Reuters Micromedex Clinical Evidence Solutions. Thomson Reuters; c2011. RED BOOK Drug References; c2011 (cited 20 January 2014); (2) Lexicomp Online™, Drug Information™, Hudson, Ohio: Lexi-Comp, Inc. 2014; 20 January 2014.

## 6. Conclusions

Type 2 diabetes is a progressive disease and the benefits derived from obtaining optimum glycemic control drives its management. In most patients, adding anti-hyperglycemic agent(s) to life style intervention is the first line of therapy. In obese patients with type 2 diabetes, it is often a challenge choosing the right agent to avoid further increases in weight. Advances in therapies both for diabetes and obesity provide clinicians with treatment that target pathways affecting glucose homeostasis, satiety and weight. It is important to recognize the adverse weight gain caused by many agents prescribed for diabetes, and to guide treatments such that we can have beneficial impacts on both glucose outcomes and weight.
